# Aesthetic Rehabilitation of a Severe Dental Fluorosis Case with Ceramic Veneers: A Step-by-Step Guide

**DOI:** 10.1155/2018/4063165

**Published:** 2018-06-06

**Authors:** Aminah M. El Mourad

**Affiliations:** Department of Restorative Dental Sciences, King Saud University, Riyadh, Saudi Arabia

## Abstract

The selection of an appropriate treatment plan for cases of dental fluorosis depends on the severity of the condition. Ceramic veneers are considered the treatment of choice for moderate to severe cases of fluorosis given the optimum aesthetics, wear resistance, biocompatibility, and long-term results of these veneers. This case report describes a step-by-step rehabilitation of fluorosed teeth, using ceramic veneers in a 26-year-old Yemeni male. The patient presented at the restorative dentistry clinics at King Saud University complaining of an unpleasant smile and generalized tooth discoloration.

## 1. Introduction

Dental fluorosis is a tooth malformation characterized by outer hypermineralization and subsurface hypomineralization, and it is caused by the chronic ingestion of fluoride during tooth development [1].

Numerous studies have reported that water fluoridation is a safe and effective public health measure for reducing the occurrence of dental caries [2, 3]. However, excessive fluoride in drinking water, exceeding a concentration of 0.5–1.5 mg/l, can lead to metabolic alteration in ameloblasts; this results in a defective matrix and improper calcification of teeth, known as dental fluorosis [4].

Dental fluorosis becomes a cosmetic concern particularly if it affects the anterior teeth. Although the causes and characteristics of dental fluorosis have been widely reported, fewer studies have discussed the proper treatment of fluorosed teeth. The selection of an appropriate treatment plan depends on the severity of fluorosis [5]. Bleaching and microabrasion have been recommended for treating mild cases of fluorosis; however, in moderate to severe cases, bleaching and microabrasion are either ineffective or may lead to only transient improvement [6], while composite restorations are prone to discoloration, chipping, and debonding. Ceramic veneers are the restoration of choice for moderate to severe cases of fluorosis given their color maintainability, wear resistance, and biocompatibility [7].

This case report presents a step-by-step aesthetic rehabilitation of a patient with severe fluorosis by using ceramic veneers.

## 2. Case Report

A 26-year-old Yemeni male patient from Taiz Province was referred to the restorative dental clinics at King Saud University, Saudi Arabia. His chief complaint was an unpleasant smile caused by generalized tooth discoloration. His medical history was irrelevant. The fluoride level in the water around Taiz Province is >3.6 mg/l [8].

### 2.1. Clinical Examination

**Figure 1:**
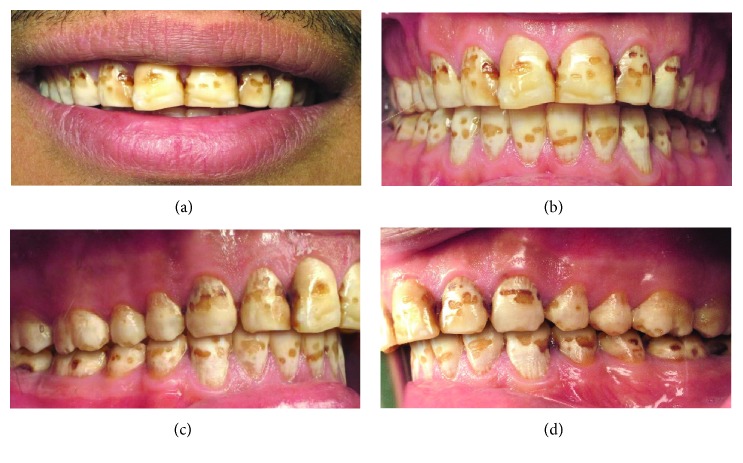
Preoperative clinical photographs: (a) smile, (b) frontal view, (c) lateral view: right side, and (d) lateral view: left side.

Clinical examination revealed generalized fluorosis with loss of the outermost enamel in irregular areas involving less than half of the entire surface, as well as changes in the morphology caused by merging pits and marked attrition (Figure 1). In this case, based on the Thylstrup and Fejerskov index (TFI) for dental fluorosis classification, the dental fluorosis was classified as TFI = 7 [9].

### 2.2. Treatment Plan

After the clinical examination, radiographs, preoperative photographs, and upper and lower alginate impressions for diagnostic models were taken. The patient was presented with treatment options, which included ceramic or composite veneers, along with the advantages and disadvantages of each option. The patient agreed to smile enhancement using ceramic veneers for his upper teeth given that he desired an optimum aesthetic and a long-term result. The veneers would be placed on the patient's upper teeth, from his upper right 2nd premolar to upper left 2nd premolar. The patient decided to postpone veneering his lower teeth, given his limited financial capacity. Diagnostic models were analyzed to evaluate the occlusion, and a diagnostic wax-up was made of white-colored wax. The use of the wax-up allows the patient to preview the desired appearance of his teeth, and this wax-up is also essential for the fabrication of a clear matrix for temporary restorations.

### 2.3. Tooth Preparation

**Figure 2:**
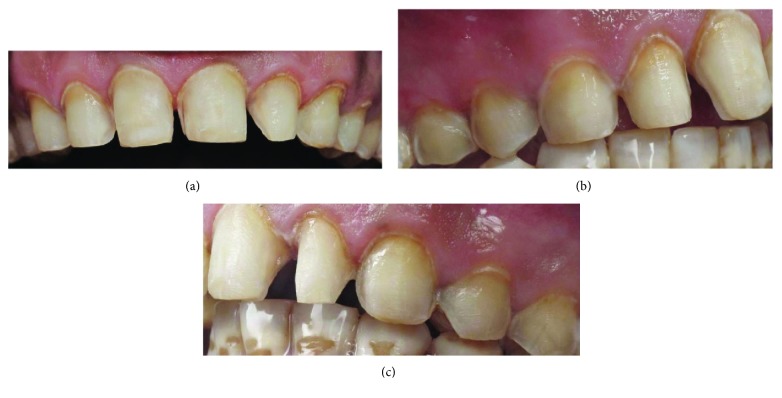
Maxillary tooth preparation: (a) frontal view, (b) lateral view: right side, and (c) lateral view: left side.

The desired shade was selected using the VITAPAN classical shade guide (VITA Zahnfabrik, Germany). The enamel of the eight maxillary teeth was prepared using a flat-end tapered diamond bur to a depth of 0.5–0.75 mm facial reduction with 1.5 mm incisal reduction (Figure 2). A chamfer finish line was maintained at the level of the gingival margin. The proximal margin was extended into the facial and gingival embrasures.

### 2.4. Final Impression and Temporization

**Figure 3:**
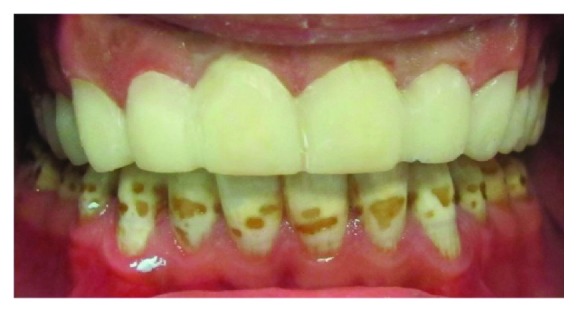
Temporary veneers.

Following tooth preparation, gingival retraction was achieved using retraction cords (Ultrapak Cord #00, Ultradent Products Inc., South Jordan, UT, USA) soaked in a hemostatic agent. Impressions were taken with a polyvinylsiloxane material (*Virtual*, Ivoclar Vivadent, Amherst, NY). The impression material was manipulated according to the manufacturer's instructions. Temporization was performed by spot etching on the facial surface of each prepared tooth with 37% phosphoric acid (Total Etch, Ivoclar Vivadent, Schaan, Liechtenstein). Bonding agent (OptiBond Solo Plus, Kerr, Orange, CA, USA) was applied on the enamel-etched spots and light cured for 20 seconds using a high-intensity light-emitting diode (LED) curing light (Elipar S10, 3M ESPE, MN, USA). The clear matrix that was previously fabricated was loaded with a temporization material (Protemp Plus, 3M ESPE, MN, USA) and placed over the prepared teeth. Light curing was done for 10 seconds per tooth. Then, the matrix was gently teased away from the prepared teeth. A number 12 scalpel blade was used to remove the partially cured temporization material. Facial and lingual embrasures were refined with a thin diamond disk, the occlusion was adjusted, and the temporary restorations were polished using polishing discs and points (Figure 3).

### 2.5. Veneer Try-In and Cementation

Ceramic veneers were fabricated with a lithium disilicate-reinforced glass ceramic material (IPS e.max Press, Ivoclar Vivadent, Schaan, Liechtenstein). Temporary veneers were removed, and the teeth were cleaned using pumice. Ceramic veneers were tried-in using a transparent shade try-in paste (Variolink Veneer try-in paste, Ivoclar Vivadent, Schaan, Liechtenstein) to assess marginal adaptation and shade.

Afterwards, veneers were prepared for bonding. Fitting surfaces of the veneers were etched with hydrofluoric acid (Porcelain Etchant 9.5%, Bisco Inc., Schaumburg, IL, USA) for 60 seconds, washed under running water for another 60 seconds, and dried with an air syringe. A layer of silane coupling agent (Monobond Plus, Ivoclar Vivadent, Schaan, Liechtenstein) was applied on the veneers' fitting surfaces and gently air-dried after one minute. Then, the prepared teeth were etched using 37% phosphoric acid for 30 seconds, rinsed, and dried. A clear mylar strip was placed interproximally to prevent inadvertent bonding to the adjacent tooth and to facilitate the subsequent removal of excess resin cement in the embrasures. A layer of bonding agent (Adhese Universal, Ivoclar Vivadent, Schaan, Liechtenstein) was applied on the prepared tooth surfaces and air-thinned. Then, Heliobond (Ivoclar Vivadent, Schaan, Liechtenstein) was placed on the prepared tooth surfaces. The inner surface of the veneers was covered with light-cured resin cement (Variolink Veneer, transparent shade, Ivoclar Vivadent, Schaan, Liechtenstein). Veneers were positioned appropriately on the teeth by applying gentle pressure, following which excess resin cement was carefully removed with an explorer before light curing. Light curing was first performed for 2 seconds, and the excess resin cement was removed with a microbrush. After that, each veneer was light-cured from the facial aspect for 40 seconds and from the lingual aspect for 40 seconds. The two veneers of the central incisors were first simultaneously cemented. This was followed by cementation of the veneers of the two lateral incisors. Then, the veneers of the two canines were cemented. Finally, veneers for the first and second premolars were cemented simultaneously on each side.

**Figure 4:**
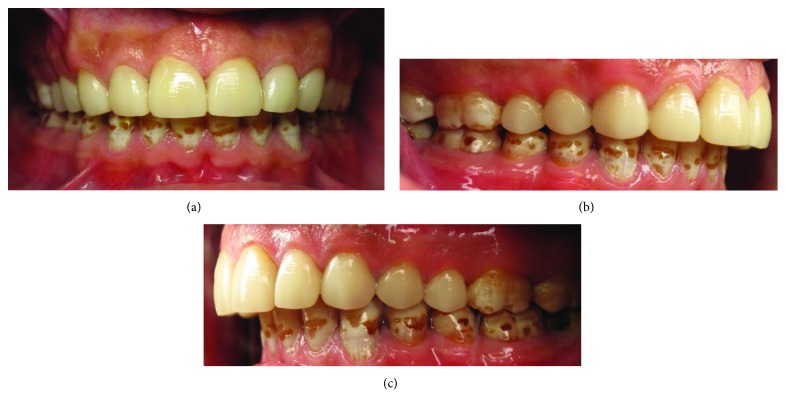
Postoperative clinical photographs: (a) frontal view, (b) lateral view: right side, and (c) lateral view: left side.

**Figure 5:**
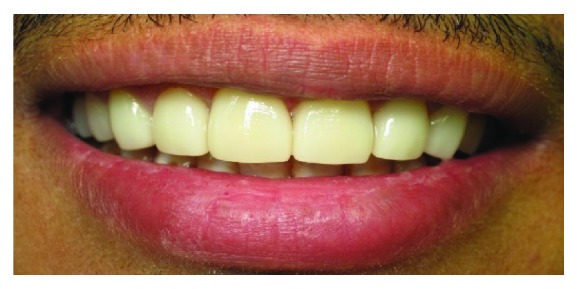
Final result.

Minimal gingival flash of the resin luting cement was removed with a number 12 scalpel blade. A flame-shaped fine diamond bur was used to finish the ceramic margins and to contour the embrasure surfaces. Occlusion was assessed and adjusted. Flossing was performed to ensure interproximal contact patency. Ceramic polishing was performed using a series of polishing cups and points (OptraFine polishing system, Ivoclar Vivadent, Schaan, Liechtenstein). Interproximal contacts were finished with finishing and polishing strips. Final surface lustre was achieved by using a diamond polishing paste with a rubber prophylaxis cup. The postoperative clinical photographs are shown in Figure 4. The patient was satisfied with the final result (Figure 5).

## 3. Discussion

The aim of the treatment in this case was to improve the patient's smile and aesthetic rehabilitation of teeth. This goal was achieved using ceramic veneers, which are the treatment of choice to mask tooth discoloration in cases of moderate to severe fluorosis.

Ceramic veneers can completely mask the discolored tooth with minimal reduction of sound tooth substance because they require a minimally invasive design preparation. In addition, advances in ceramic materials have facilitated this process. Ceramic veneers provide both predictable and long-lasting aesthetic rehabilitation [10, 11].

The durability and clinical success of porcelain veneers have been widely investigated in the literature. It has been reported that ceramic veneers provide durable and successful restoration with an estimated survival probability of 93.5% over 10 years [12]. Satisfactory results were obtained in a case of fluorosed teeth restored with porcelain laminate veneers over a 6-year follow-up [13]. Furthermore, numerous studies have demonstrated acceptable aesthetic outcomes in cases of moderate to severe fluorosis where restoration with porcelain veneers was performed [14].

## 4. Conclusion

Ceramic veneers are considered one of the most popular restorative materials in aesthetic dentistry. They provide excellent aesthetic results when an appropriate treatment plan and protocol are used during the clinical and laboratory fabrication stages. This case report describes the use of ceramic veneers to enhance the appearance of fluorosed teeth, thus improving the patient's smile and, consequently, self-esteem.
